# First Genetic Evidence Confirming the Occurrence of the Siberian Chiffchaff *Phylloscopus collybita tristis* Blyth, 1843, in Italy

**DOI:** 10.3390/ani16192986

**Published:** 2026-09-23

**Authors:** Gea Manganaro, Filippo Tatino, Alessandro Motta, Giada Santa Calogero, Venera Ferrito, Anna Maria Pappalardo

**Affiliations:** 1 Section of Animal Biology “M. La Greca”, Department of Biological, Geological and Environmental Sciences, University of Catania, Via Androne 81, 95124 Catania, Sicily, Italy; gea.manganaro@phd.unict.it (G.M.); giada.calogero@phd.unict.it (G.S.C.); vferrito@unict.it (V.F.); 2 A.S.O.I.M. Associazione Studi Ornitologici Italia Meridionale OdV, Via Cavalli di Bronzo 95, 80046 San Giorgio a Cremano, Naples, Italy; filippo.tatino@gmail.com (F.T.); alessandromotta.vet@gmail.com (A.M.)

**Keywords:** Siberian Chiffchaff, *Phylloscopus collybita tristis*, chiffchaff complex, genetics, subspecies, cryptic taxa

## Abstract

The Siberian Chiffchaff is a subspecies within the “Chiffchaff complex”, characterized by a typically pale greyish plumage and distinctive vocalizations, although reliable identification may still require genetic confirmation. In Italy, the taxon is considered fairly rare and poorly documented, with previous records based exclusively on identifications provided from registrations of the call or photos. This study provides the first genetic confirmation of the occurrence of Siberian Chiffchaff in Italy through molecular analysis of an individual captured at the Longola ringing station (Poggiomarino, Naples) on 21 December 2025. DNA was extracted from a feather sample. A fragment of NADH dehydrogenase 2 mitochondrial gene and a fragment of Aconitase 1 intron 9 were amplified and sequenced. A phylogenetic dataset including representatives of all taxa within the “Chiffchaff complex” was assembled and analyzed using maximum likelihood methods. The resulting phylogeny placed the sampled individual within the *P. c. tristis* clade. Morphological examination of photographic material also supported the identification, highlighting the almost complete absence of green and yellow tones in the plumage. Among 452 Chiffchaffs captured at the study site, between October 2025 and March 2026, only one individual showed potentially compatible tristis-like phenotypic traits and was genetically confirmed.

## Simple Summary

The Siberian Chiffchaff is a small migratory bird that can be difficult to distinguish from closely related chiffchaffs. In Italy, its status is still poorly understood, and previous records were based only on photographs or sound recordings. In this study, we investigated the identity of a suspected Siberian Chiffchaff captured in a bird ringing station in southern Italy in December 2025. Genetic analyses confirmed that the individual belonged to the Siberian Chiffchaff subspecies, supporting the photographic material. Among 452 chiffchaffs examined at the study site during the autumn and winter season, only one bird showed characteristics consistent with the Siberian Chiffchaff and was genetically confirmed. This finding provides the first genetic confirmation of the Siberian Chiffchaff in Italy and demonstrates the value of combining field observations with genetic analyses when studying birds that are difficult to identify. Improved identification of rare migratory birds contributes to a better understanding of their distribution, movements, and occurrence patterns, helping researchers to assess their status more accurately across Europe.

## 1. Introduction

The Siberian Chiffchaff *Phylloscopus collybita tristis* Blyth, 1843 is one of the six subspecies of *P. collybita* (Vieillot, 1817) and, together with other nine taxa, belongs to the cryptic and polytypic “Chiffchaff complex” [1]. It is characterized by a distinctive pale grayish plumage [2] and marked bioacoustics differences [3] which may facilitate the identification in the field, whereas genetic analyses remain the only reliable method for unequivocal identification of all the other *P. collybita* subspecies. The breeding range of this subspecies extends from the Kolyma River in eastern Siberia to the western foothills of the Urals [4], where it encounters populations of the Scandinavian Chiffchaff *P. c. abietinus* in an overlapping area where individuals can show intermediate phenotypes [5]. The wintering range is mainly extended to the Indian subcontinent, marginally in the eastern part of the Middle East [6] and in western Europe [7]. The subspecies also occurs in Italy with records dating back to 1976 during migration and wintering periods [8].

The aim of this study is to report the first genetically confirmed occurrence of the Siberian Chiffchaff in Italy with a phylogeny based on the analysis of NADH dehydrogenase 2 (*ND2*) mitochondrial gene from a mostly grayish individual captured at Longola ringing station in Poggiomarino (Naples, Italy). Considering the presence of hybridization zones between the subspecies *P. c. tristis* and *P. c. abietinus*, an additional analysis was conducted using the nuclear Z-linked marker Aconitase 1 intron 9 (*ACO1I9*) to obtain information on the paternal lineage of the individual.

## 2. Materials and Methods

DNA was obtained from a single feather that was accidentally lost during the handling of the individual at the Longola ringing station on 21 December 2025. The individual was ringed, measured, and its general physical condition was assessed through fat and muscle scores, according to standard ringing protocols [9,10]. Age was determined on the basis of moult stage. The individual was subsequently photographed and then immediately released.

The feather sample was stored in a 96% ethanol solution at 4 °C until laboratory analyses were performed. The sample was labelled “LPP_9V0253”. Molecular analyses were conducted starting from the DNA extraction from calamus [11] using a QIAGEN DNeasy Blood & Tissue Kit (Qiagen, Hilden, Germany) and following a standard extraction protocol from animal tissues. PCR was carried out with a starting cycle at 95 °C for 3 min, followed by 35 cycles at 95 °C for 45 s, 57 °C for 45 s, 72 °C for one minute, and a further elongation cycle at 72 °C for 10 min. Specific primers for the amplification of *ND2* mitochondrial sequence were chosen: L5219Met (Forward) and H6313Trp (Reverse) [12,13]. The obtained amplicons were sequenced at Eurofins Genomics, and the resulting sequence was submitted to GenBank [14] with accession number PZ462782. A dataset of 46 sequences was assembled including the sequence of the individual obtained in this study, including five sequences from each taxon of the “Chiffchaff complex” from Raković et al. [15]. Sequence alignment was performed using MAFFT version 7.4 [16] and subsequently edited through Aliview version 1.30 [17]; in particular, the terminal regions of the sequences were trimmed to ensure that all sequences had the same length. The alignment was then used to construct a haplotype network in Hapsolutely v0.2.3 [18] using the TCS algorithm. The resulting network was subsequently graphically edited in Inkscape v1.4.

Nuclear DNA analyses included molecular sexing of the individual, which was performed following the standard amplification protocol for CHD1F and CHD1R primers [19]. The result was subsequently visualized through the electrophoretic analysis.

In addition, the nuclear Z-linked marker *ACO1I9* was amplified using specific primers and PCR protocol [20]. The amplicons were sequenced at Eurofins Genomics and the resulting sequence was submitted to GenBank with accession number (accession number will be included before publication). The obtained sequence was then aligned with 165 *ACO1I9* sequences from *P. c. abietinus* and *P. c. tristis* retrieved from GenBank. The multiple sequence alignment was carried out through MAFFT version 7.4 and then inspected and trimmed in Aliview. The alignment was then used to construct a haplotype network in Hapsolutely v0.2.3 [18] using the TCS algorithm. The resulting network was subsequently graphically edited in Inkscape v1.4.

## 3. Results

During ringing operations, all the biometric parameters of the individual were recorded and are reported below together with two photos (Figure 1). The age was estimated through the examination of moult stage and classified as 3, indicating that a juvenile individual born in the same year as capture (2025). Fat score was visually assessed as 4 on a scale from 0 to 8, where 0 indicated a total absence of visible fat and 8 indicated extensive fat accumulation. Muscle score was evaluated by palpation and assessed as 2 on a scale from 0 to 3, where 0 corresponded to severe muscle atrophy and 3 to well-developed musculature.

The individual firstly captured on 21 December 2025 was subsequently recaptured on 31 January 2026.

Regarding the mtDNA marker analysis, the alignment edited through Aliview resulted in a set of 46 partial *ND2* sequences of 960 bp for a total of 35 haplotypes.

The TCS haplotype network showed the placement of the target individual within the *P. c. tristis* haplogroup (Figure 2).

Molecular sexing confirmed that the individual was female, as the electrophoretic analysis revealed the presence of two fluorescent bands.

The alignment of the nuclear marker dataset was edited in Aliview, resulting in a final set of 166 partial ACO1I9 sequences of 289 bp. The analysis in Hapsolutely produced a haplotype network (Figure 3) showing the genetic relationship between 14 haplotypes and the position of the single haplotype obtained in the individual analysed in the present study in relation to those previously reported in the literature for the two subspecies *P. c. abietinus* and *P. c. tristis*. Among the 14 haplotypes identified, one was found to be widely shared between the two subspecies.

## 4. Discussion

The Siberian Chiffchaff appears to be the most distinct among the *P. collybita* subspecies, exhibiting a characteristic pale greyish plumage and markedly different vocalizations. Genetic evidence further supports the differentiation suggested by analyses based on phenotypic traits so much that some authors consider it a full species [6]. However, the extensive hybridization occurring between *P. c. tristis* and *P. c. abietinus* [21], and the resulting gene flow, precludes the split of the two taxa. Despite these significant differences, the correct field identification of the Siberian Chiffchaff remains challenging. The subspecies is considered fairly rare in Italy, especially during the winter, and even though it appears to occur more regularly during migration periods, precise data on its frequency across the country are lacking. Furthermore, both the nominate subspecies and *P. c. abietinus*, which are certainly the most widespread in Italy, show considerable intraspecific variation in the plumage coloration, with an extremely variable amount of yellow and green tones. Moreover, individuals belonging to North European populations may also appear very pale [2]. A recent study on the occurrence of different Chiffchaff subspecies in eastern Sicily showed that individuals genetically confirmed as *P. c. abietinus* were considerably more similar to *P. c. collybita* than previously expected, appearing virtually indistinguishable [22]. Similar results were reported in a previous study in the Netherlands [23,24]. Nevertheless, given the intraspecific variability within the Chiffchaffs complex, many individuals may exhibit ambiguous phenotypic traits, making reliable identification difficult. Out of 452 Chiffchaffs captured at Longola ringing station between October 2025 and March 2026, only the individual LPP_9V0253 was confirmed as *P. c. tristis* on the basis of both photographic and mtDNA evidence. This individual did not produce any call during ringing operations that could have helped with the identification. Moreover, from a biometric perspective, it is not possible to distinguish *P. c. tristis* from the other subspecies. Wing measurements are some of the most used morphometric characters for discriminating between closely related warbler taxa; for example, they are useful to distinguish the Willow Warbler *Phylloscopus trochilus* from the similar Common Chiffchaff *Phylloscopus collybita*. Unfortunately, at the subspecific level, there is a broad range of overlap in measurements, meaning that these characters are not sufficiently reliable for identification [10]. Conversely, plumage coloration is a highly informative phenotypic trait for the identification of the Siberian Chiffchaff. In fact, some specific traits have been examined from the analysis of photographical material, revealing an almost total absence of green tones in the upper parts and a pale yellow-lacking coloration of supercilium, ear-coverts, side of neck and breast. These phenotypic traits already strongly suggested assignment to the subspecies *P. c. tristis*, which was subsequently confirmed by a phylogenetic analysis based on *ND2* mitochondrial marker, one of the most widely used genetic markers in Chiffchaffs studies.

Considering the documented hybridization between the subspecies *P. c. tristis* and *P. c. abietinus*, the possibility that the individual analyzed in this study is in fact a hybrid between the two subspecies cannot be completely excluded. The mitochondrial genome is maternally inherited; therefore, based on the results obtained from the *ND2* marker analysis, the individual could potentially be the offspring of a *tristis* female and an *abietinus* male. For this reason, and to provide a more comprehensive investigation, a fragment of the nuclear marker *ACO1I9* was also analyzed. This marker corresponds to an intronic sequence located on the Z sex chromosome. The individual analyzed in this study is a female, which in birds represents the heterogametic sex (ZW), whereas males are the homogametic sex (ZZ). Therefore, the analysis of a Z-linked marker is particularly useful in this case, as it can provide genetic information associated with the paternal lineage.

The analysis produced a haplotype network showing the position of the target individual’s haplotype relative to those of the two subspecies involved in documented hybridization events (*P. c. abietinus* and *P. c. tristis*). However, the node corresponding to the target individual was connected to a haplotype shared between the two subspecies. By examining the study by Rakovic et al. [15], it was possible to verify that their results included two haplotypes widely shared among individuals belonging to both subspecies. These shared haplotypes were observed both in populations from geographically distant areas, such as *abietinus* from Sweden and *tristis* from Siberia, and in populations from the Ural region, where hybridization between the two subspecies is known to occur. Therefore, this analysis alone does not allow us to completely exclude the possibility that the target individual represents an *abietinus/tristis* hybrid.

## 5. Conclusions

The result obtained in this study represents the first genetically confirmed record of the Siberian Chiffchaff in Italy. The mtDNA analysis clearly placed the target individual into the *tristis* haplogroup, even though the possibility that it represents a hybrid between *abitetinus* and *tristis* cannot be completely excluded based on the results of the nuclear DNA marker analysis.

The identification of species and subspecies belonging to the Chiffchaff complex may be challenging even in the hand. The result obtained in this study highlights the importance of integrating bird ringing activities with molecular analyses, particularly in intricate cases such as this one. Moreover, it emphasizes the value of genetic studies in the investigation of Siberian Chiffchaff, as a rare and cryptic taxon, whose status in Italy remains still poorly understood. Studies such as this, conducted on a larger scale and combined with the collection of records supported by bioacoustic evidence, could help clarify the nature of the occurrence of the Siberian Chiffchaff in Italy in the future.

## Figures and Tables

**Figure 1:**
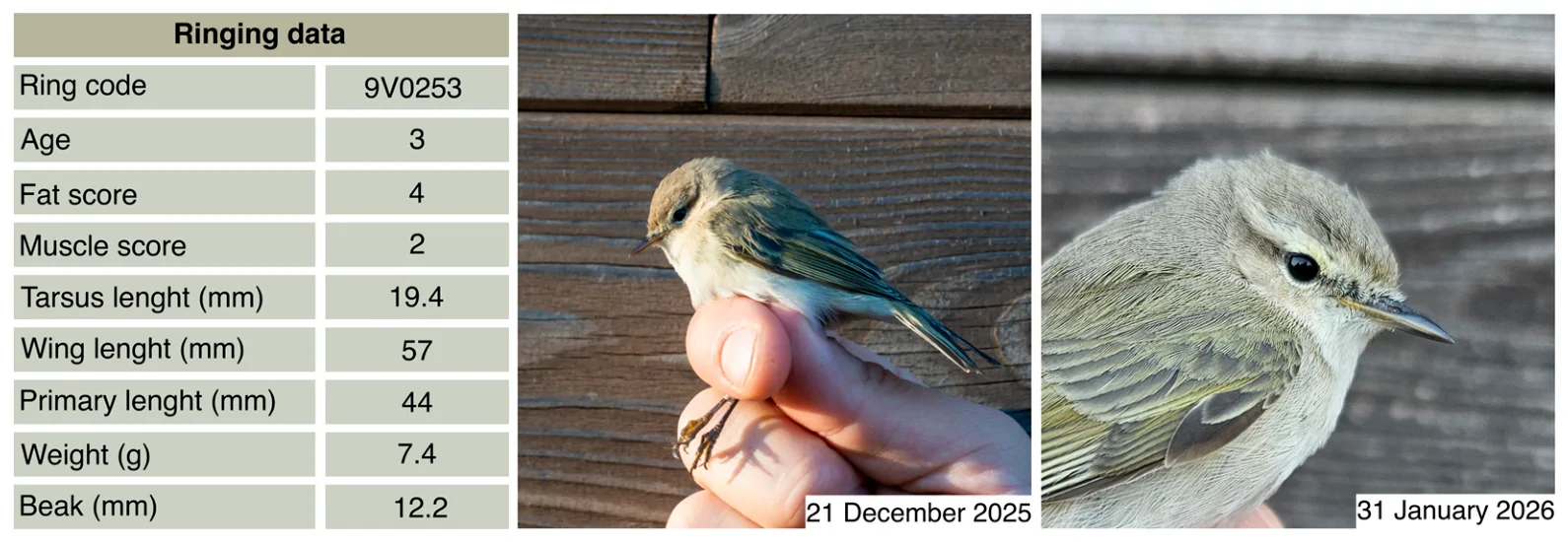
Ringing data and photos of the target individual. Photo of 21 December 2025 showing the general appearance. Photo of 31 January 2026 showing a focus on the face. Photos taken by Filippo Tatino and Alessandro Motta. Plate realized through Inkscape.

**Figure 2:**
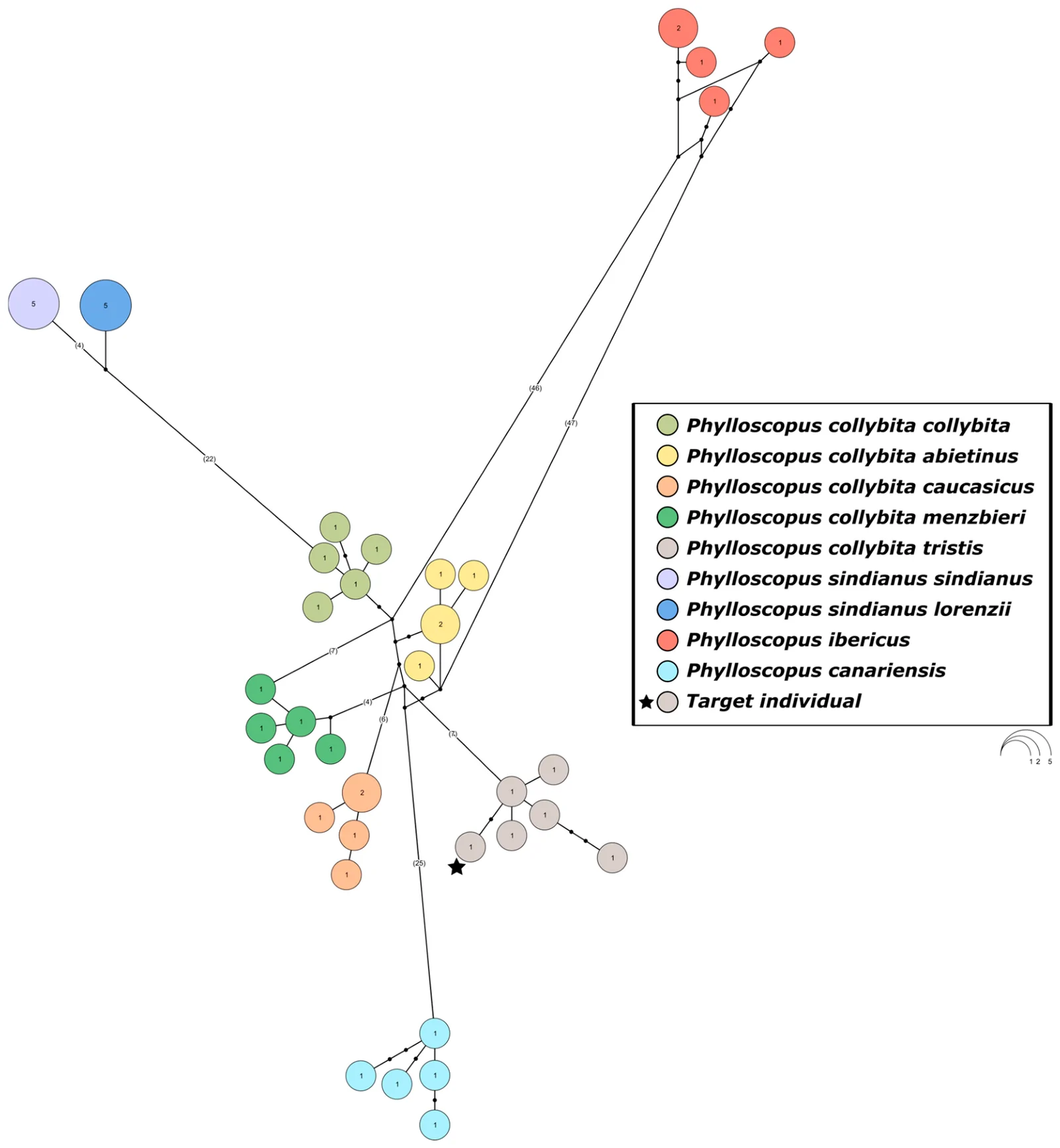
Haplotypes network showing 35 *ND2* haplotypes species and subspecies of *Phylloscopus* genus. Figure realized through Inkscape. The black dots represent the unsampled haplotypes. Numbers in bracket indicate the mutational steps.

**Figure 3:**
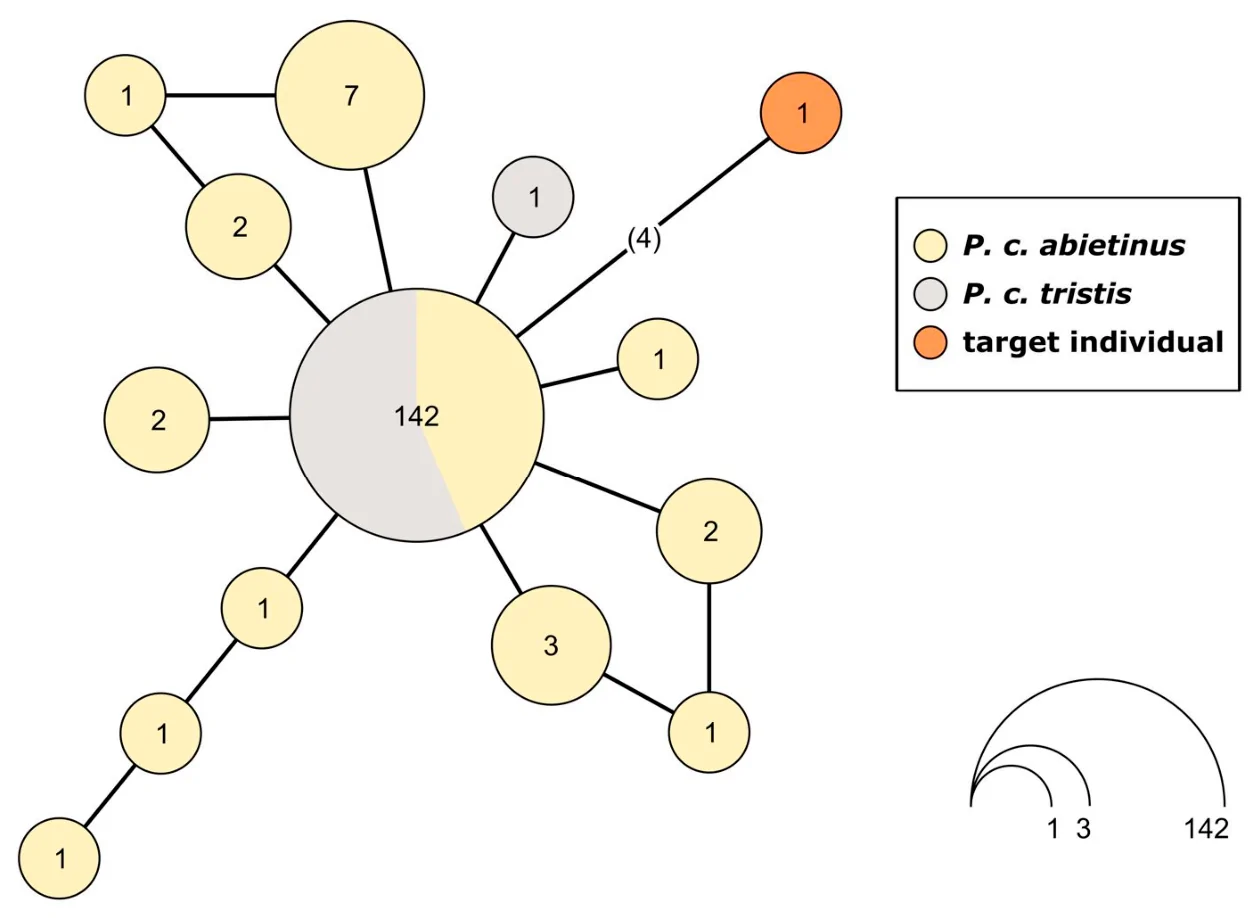
Haplotypes network showing 14 haplotypes of partial Aconitase 1 introne 9 (*ACO1I9*) nuclear marker of the two subspecies *P. c. abietinus* and *P. c. tristis*. Figure realized through Inkscape. Numbers in bracket indicate the mutational steps.

## Data Availability

The molecular data of the sequence are available in GenBank. All the other data presented in this study are available on request from the corresponding author.
